# Legal themes concerning obesity regulation in the United States: Theory and practice

**DOI:** 10.1186/1743-8462-5-14

**Published:** 2008-06-25

**Authors:** James G Hodge, Andrea M Garcia, Supriya Shah

**Affiliations:** 1 Associate Professor, Johns Hopkins Bloomberg School of Public Health; Executive Director, Centers for Law and the Public's Health: A Collaborative, Baltimore, MD, USA; 2 Policy Analyst, Division of Medicine and Public Health, American Medical Association Chicago, IL, USA; 3 Research Intern, Center for Law and the Public's Health: A Collaborative, Baltimore, MD, USA

## Abstract

Despite national health objectives to reduce the incidence of obesity to 15% of the population by 2010, public health data suggest that the incidence of obesity in the United States is actually increasing. The U.S. recognizes that it (like other industrialized countries) faces an epidemic of obesity and related health conditions. How can U.S. jurisdictions (federal, state, and local) and the private sector respond to this epidemic through laws and policies that are directly or indirectly designed to address obesity? This article analyzes the theoretical and practical roles of law as a tool to curb obesity in the U.S. It proffers ten major legal themes to address obesity among the U.S. population, including: (1) use of incentives to encourage healthier behaviors; (2) use of financial disincentives to discourage unhealthy behaviors; (3) requirements to improve food quality, diversity, or availability; (4) compensation for injured persons seeking recourse; (5) restriction of access to unhealthy foods; (6) regulations aimed at influencing consumer choices; (7) control of marketing and advertising; (8) creation of communities that support healthy lifestyles; (9) physical education/fitness requirements; and (10) insurance coverage mandates.

## Background

Obesity among populations in industrialized countries is one of the most serious public health threats of the modern era. Although many developed countries, including Australia and New Zealand, face this public health dilemma, the United States population actually has the highest prevalence of obesity among developed nations [1]. Due to its health impact and costs in terms of health care expenditures and loss of productivity, reducing obesity in child and adult populations is among the nation's priority health goals for the Twenty-first century. Achieving this goal is, however, challenging. Recent national surveillance data indicate that 17.1% of children and adolescents (age 2–19 years) are overweight [2]. Between 1988–1994 and 2003–2004, the percentage of those categorized as overweight increased from 7.2% to 13.9% among 2–5 year olds, from 11% to 19% among 6–11 year olds, and from 11% to 17% among adolescents in the U.S. [3]. The prevalence of adult obesity (those over 20 years of age) has burgeoned to 23% of the American population in 2003–2004 [4]. These trends suggest that Americans are getting heavier despite concerted efforts to address obesity as a public health problem in the U.S. A national goal set in 2000 of reducing childhood and adolescent overweight or obesity from 11% to 5%, and adult obesity from 23% to 15%, by 2010 is now virtually impossible to achieve [5]. Recently, a survey conducted by researchers at the Johns Hopkins Bloomberg School of Public Health estimated that by 2015, potentially 41% of American adults will be obese, and 24% of children and adolescents will be overweight or obese [6].

Improved surveillance of obesity among industrialized populations globally has led government and private sector authorities to address obesity as a public health crisis. Public attitudes and behaviors that have contributed to a lifestyle that leads to obesity are quickly changing. Most Americans now consider obesity a serious individual and communal health problem. Public health agencies in the U.S. are working to strategize and develop new approaches to curb obesity. The private sector is contributing resources to combat this problem. Recently, the Robert Wood Johnson Foundation announced a U.S. $500 million program to address obesity, particularly among children. Underlying these public and private sector efforts are a host of legal responses at each level of government (federal, state, and local) in the U.S. that are designed to directly or indirectly address obesity and the factors that contribute to it as a public health problem. In this Commentary, we present 10 major legal themes of obesity regulation in the U.S., and offer examples of how laws reflect these themes domestically.

## Legal Themes to Regulate Obesity in the United States

### Use of Incentives to Encourage Healthier Behaviors

Through the legal use of incentives, government is able to encourage and promote healthier behaviors, particularly more nutritious diets and the need for increased physical activity, among citizens. For example, the state of California has developed a Local Incentive Awards Program, which qualifies local public health agencies for federal matching funds to develop nutrition education and physical activity promotion interventions for low-income communities [7]. The state legislature of New Jersey has enacted legislation that more directly provides incentives for its citizens to engage in healthy behaviors by granting tax deductions as a reward for biking to work [8]. One proposed incentive, which could potentially impact all states, involves the U.S. Medicaid program. This federal-state partnership program provides low-income and other at risk populations with access to basic health care services through subsidized health insurance. Some states, including Florida [9], Iowa [10], and Kentucky [11], have requested and received waivers from federal restrictions on the use of federal funds via the Medicaid program (which provides basic health insurance and services to mostly low income citizens) to experiment with healthy behavior incentives.

Florida's "Enhanced Benefits Account" program allows Medicaid beneficiaries to earn credits for engaging in healthy behaviors. Enrollees can later use these credits to purchase health-related items (e.g., pharmaceuticals, medical devices) that they might otherwise have to expend their own resources out-of-pocket [12]. Medicaid managed care organizations (MCOs) have developed policies that offer their own incentives, such as offering members gifts for responding to outreach programs [13].

Another example of an incentive-based initiative is the U.S. food stamp program. Food stamps provide recipients with less than U.S. $80 per month on average to help them purchase foods of their choice [14]. A 2004 study presented at the American Heart Association found that "a family of four would need to spend U.S. $227 a month in excess of food stamp benefits to make heart-healthy foods part of their daily diet" [15]. As a result, states are exploring ways to encourage the purchase of healthy products through food stamps. California, for example, has passed legislation to increase fruit and vegetable consumption by providing a specified bonus for every dollar of food stamps spent on fresh produce [16,17]. These types of incentive programs illustrate how government and private sector entities can use laws and policies to encourage populations to engage in healthier behaviors.

### Use of Financial Disincentives to Discourage Unhealthy Behaviors

A counter strategy to providing incentives to encourage healthy behaviors is to use law, particularly taxation, as a tool to discourage unhealthy behaviors. Taxation has historically been used effectively to control the consumption of alcohol and tobacco products through federal, state, and local taxes. Despite the addictive nature of tobacco products, studies indicate that tobacco consumption declines an average of 4% for every 10% increase in price [18]. Building on these results, 17 states and the U.S. District of Columbia have placed specific taxes (either through sales or excise taxes) on foods and/or beverages of low nutritional value [19]. The idea is that these taxes will provide a strong disincentive for individuals against purchasing and ingesting these products, resulting in a reduction in obesity counts. For those who still choose to purchase these products, tax revenues from sales could be used to fund healthy eating or obesity prevention campaigns [20]. California's 7.25% sales tax on soft drinks, for example, generates about U.S. $218 million in general revenues annually [20]. Unfortunately, among those states that currently feature these taxes, very few earmark the funds for public health campaigns to address obesity [20]. Opponents of the taxes say that they are regressive and are unlikely to encourage people to consume healthier foods [19].

### Requirements to Improve Food Quality, Diversity, or Availability

Government's ability to require improvements in nutrition applies to multiple settings where people obtain food. An obvious example where the government directly sets food standards is in public schools. Under the U.S. Department of Agriculture's (USDA) Food and Nutrition Service, which administers the National School Lunch and Breakfast program, federal subsidies are provided to schools as long as nutrition guidelines are met [17]. However, "competitive foods" sold through vending machines, school stores, or snack bars are not regulated under federal nutrition standards [17]. As a result, the U.S. Congress commissioned the Institute of Medicine (IOM) to develop a set of standards for these foods [21]. Released in April 2007, the IOM report concluded that the sale of competitive foods should be limited in schools. When these foods are available, however, they should be consistent with the 2005 Dietary Guidelines for Americans [21]. Putting these recommendations into practice would involve changes in federal, state and local laws and school policies. The U.S. Senate is considering the Child Nutrition Promotion and School Lunch Protection Act, which would give the USDA the authority to regulate competitive foods and penalize non-compliant schools [22].

In addition, threats of sanction or government regulation may result in industry self-regulation. Consider the 2006 voluntary agreement signed by soft drink giants Coca-Cola, Pepsi, and Cadbury Schweppes, to remove high-calorie sodas from schools by 2009 [23]. A 2003 lawsuit filed in California against the U.S. multi-conglomerate Kraft Foods sought to prevent the marketing and selling of Oreo brand cookies, which contained trans fats, to children in the state. The lawsuit was dropped when Kraft announced that it was working to reduce trans fat in its cookies and agreed to cease all in-school marketing [24].

### Compensation for Injured Persons Seeking Recourse

Litigation in the pursuit of some sort of compensation for obese consumers is increasingly seen as a viable option in the U.S. Cases have been brought against the food industry claiming that it engaged in deceptive practices, inadequately disclosed health risks, or mislead consumers through its advertisements [25]. For example, in 2002, a class action lawsuit was filed against Robert's American Gourmet Food, Inc. and Keystone Food Products for distributing and manufacturing products, under the names Pirate Booty, Veggie Booty and Fruity Booty, which had a substantially higher fat and caloric content than advertised [26]. Allegations of fraud and deceptive trade practices resulted in a judgment of U.S. $3.5 million and U.S. $790,000 in attorney's fees. On appeal, however, the class was effectively decertified, and the lower court's judgments were ultimately dismissed [27].

A widely-reported case of persons seeking compensation for obesity-related injuries is *Pelman v. McDonald's Corp*. In 2002, the parents of two obese minors filed a complaint against McDonald's Corporation in New York State alleging deceptive practices, negligence, and failure to warn consumers of the harms of ingesting food at McDonald's restaurants [28]. This case was initially dismissed, as was an amended complaint suggesting that the alleged deceptive practices were in violation of state consumer protection laws [29]. The case appeared closed until the parents appealed the second dismissal and won [30]. The case is moving forward to trial on allegations that McDonald's created the false impression that its food products were nutritionally beneficial [31].

To counter the proliferation of obesity-related lawsuits against food manufacturers, distributors, marketers, advertisers, sellers and trade associations, Congress considered, but did not pass, the Personal Responsibility in Food Consumption Act and Commonsense Consumption Act in 2003 [32,33] and 2005 [34,35]. Collectively, these bills sought to protect food manufacturers and retailers from civil actions brought by obese consumers, deferring to executive agencies (and not the courts) to determine appropriate measures to respond to industry practices. While such measures have failed at the federal level, nearly half of American states have enacted "personal responsibility" laws that shield fast food companies from obesity-related tort claims [19].

### Restricting Access to Unhealthy Foods

Restricting access to unhealthy foods through governmental zoning laws and the voluntary action of restaurants can presumably lead to improvements in human nutrition by limiting the availability of detrimental foods and increasing access to more wholesome food choices.

Local zoning laws in the U. S. can be used to create a healthier retail food market through several different themes [36], including (1) rezoning residential areas to restrict development to restaurants that do not serve "fast-food;" (2) providing incentives for developers to offer health food stores among commercial options; and (3) requiring fast food restaurants to offer a minimum number of healthy choices [36]. Zoning laws can justify (1) outright bans of fast food establishments, such as San Francisco's (California) prohibition of "formula retail uses" in a historic neighborhood district, and (2) restrictions, such as the city of Detroit's (Michigan) policy that certain fast food restaurants may not be built within 500 feet of a school [36]. When challenged, U.S. courts typically uphold such laws on public health and non-public health bases. For example, in the Massachusetts case of *Bellas v. Planning Board of Weymouth *(2002), an appellate court found that a drive-thru window created by a franchised doughnut shop would generate increased traffic that could affect the safety of children walking to school, and hence denied the required permit [36]. The effect of the court's decision, which was grounded in zoning laws, is to limit some access to food choices that could contribute to an increase in weight among some consumers.

Limiting access to unhealthy foods does not always require governmental interventions. Some restaurants, under pressure from lawsuits and municipal requests, have focused on modifying their recipes or products to decrease fat content, particularly trans fats. Prompted by a lawsuit filed by the Center for Science in the Public Interest (CSPI), Kentucky Fried Chicken (KFC) began using trans fat free soybean oil in all of its restaurants in 2007 [37]. In May 2005, the city of Tiburon, California became the first "trans fat free city" when its restaurants switched to using alternative oils [38]. Larger jurisdictions, including New York City and Philadelphia, PA, have since banned the use of trans fats in their restaurants [38]. Consequently, major chains such as Wendy's and Chili's have had to modify their recipes to improve the nutritional quality of their food [37].

### Regulations Aimed at Influencing Consumer Choices

Consumers need nutrition information to make healthy decisions about foods. Correspondingly, government has sought to increase regulation of nutrition and menu labels in the U.S. The federal Nutrition Labeling and Education Act (NLEA) of 1990 requires most foods offered for retail purchase to be labeled with nutrient and ingredient information, and for health claims to comply with standards [39].

Processed meals served in restaurants, however, are not currently required to include nutrition information. Some restaurants provide this information about their meals voluntarily under varying formats [40]. To provide consumers with nutrition information, menu labeling bills have been introduced at the federal, state and local levels. At the federal level, the Menu Education and Labeling Act (MELA), first proposed in 2003 [41] and reintroduced in 2006 [42], would require chain restaurants with 20 or more locations to provide consumers with information on calories, sodium, fat, and trans fat content [43]. At the state level, Maine was the first to introduce menu labeling legislation. In 2006, nine states and D.C. considered a variety of menu labeling bills. None of these bills, however, were enacted, most likely due to opposition from the restaurant industry [40]. At the local level, there has been some success in enacting these regulations, such as when the New York City Board of Health approved measures requiring restaurants to make caloric (or energy) information publicly available at the point of purchase by posting it on menus and menu boards for consumers to read before they order [44]. The New York Restaurant Association recently filed a federal lawsuit against the measures, arguing that the requirement to post this information violates their First Amendment rights and is preempted by the NLEA [45].

### Control of Marketing and Advertising

Marketing and advertising of food products can be powerful tools for influencing consumer choices, particularly among children. In 2006, the IOM reported that food and beverage marketing has an influence on the preferences and purchase requests of children and may contribute to negative diet-related health outcomes and risks [46]. In the U.S., the food industry has adopted a self-regulatory program, the Children's Advertising and Review Unit (CARU), that monitors and reviews advertising directed at children, and determines whether there has been a violation. However, action is only taken once a violation has been identified. Even then, the cooperation of advertisers is sought to make changes on a voluntary basis [47].

The U.S. Federal Communications Commission (FCC) and the Federal Trade Commission (FTC) share regulatory responsibility for advertising. The FCC regulates interstate and international communications through radio, television, wire, satellite and cable [48]. The FTC protects consumers against unfair and deceptive practices [49]. However, government's authority to regulate advertising must constantly be balanced with the industry's First Amendment right to commercial speech (e.g., speech in any form that advertises a business product, service, or purpose)[50]. Commercial speech in the United States is specifically protected from unwarranted governmental infringements via freedom of speech guarantees [51]. While government may restrict commercial advertising, its interest must be substantial, regulations must be proportional to that interest, and regulations must not be more extensive than necessary to serve that interest [51]. A recent federal report encourages increased company initiatives and a strengthened self-regulatory system to address childhood obesity [52]. The FCC Task Force on Media and Childhood Obesity is currently studying the links between advertisements, television viewing habits, and the increase in childhood obesity, and will issue a report with recommendations [53].

Consumer protection laws and litigation can also be used to restrict unhealthy advertising to children. In January 2006, the CSPI, Campaign for a Commercial-Free Childhood (CCFC), and parents indicated their intent to file a suit against a major U.S. broadcaster and breakfast cereal company for allegedly engaging in acts and practices that were unfair and deceptive in the marketing and sale of foods of poor nutritional quality to children under 8 years of age [54]. Though ultimately dropped, the lawsuit sought to stop the marketing of "food of poor nutritional quality or product line or brand for which more than 50% of the food products are of poor nutritional quality" to children [54].

### Creation of Communities that Support Healthy Lifestyles

A community's built environment, including proximity of facilities, availability of walking/biking paths, and housing density, has been shown to heavily influence people's lifestyles [55]. As a result, government officials have developed several programs that encourage healthy behaviors and promote physical activity as a way to combat ill health and obesity.

Creating communities that support healthy lifestyles means redesigning or rebuilding environments so that they offer safe opportunities for physical activities. For example, the state of Hawaii's "Bike Plan Hawaii 2003" includes a guide for improving biking facilities and monitoring biking conditions [56]. The Florida legislature enacted the "Florida Greenways and Trails System" in 2005 to establish a trail system that provides people with access to "healthful outdoor activities" [57]. Because safety is one of the major concerns preventing some children from walking or biking to school, the federal government implemented its Safe Routes to School (SRTS) Program in August 2005. This program provides funding for a wide array of municipal projects, such as the building of safer street crossings, so that walking/biking to school can become a safe and routine activity [58].

As part of the creation of comprehensive plans that combine urban planning with the promotion of physical activity, the federal Centers of Disease Control and Prevention (CDC) developed the Active Community Environments (ACES) initiative, which promotes the development of accessible recreation facilities in addition to walking/biking trails. ACES' guidebook assists public health officials in planning and promoting local recreation facilities. CDC also collaborates with entities, such as the federal Environmental Protection Agency (EPA), to assess the relationship of land to uses and survey attitudes of Americans concerning the environment, walking, and biking [55]. States have also engaged in their own campaigns to promote healthy lifestyles. The Healthy Arkansas Initiative, for example, helps citizens locate wellness resources in their community [59].

### Physical Education/Fitness Requirements

Through legislation, physical education and fitness requirements have been implemented in schools and the workplace. Offering physical education classes and programs in schools allows children to learn and develop healthy exercise habits. Illinois, Arkansas, Iowa, Kentucky, Louisiana, New Jersey, New York, and Rhode Island all require some level of physical education for elementary and secondary students [60]. Similar requirements in the workplace provide employees with incentives and opportunities to incorporate exercise into their workday, with corresponding health and economic benefits. A legislative resolution in Arkansas in 2001 requested that all directors of state agencies design and implement physical activity programs as part of their workday. The state also encourages all employers to offer "worksite wellness programs" by providing educational information and guidelines for developing incentive programs and integrating programs into the existing organizational structure [61]. The state-sponsored Healthy Hawaii Initiative promotes similar educational and physical activity projects [62].

In 2002, the federal Department of Health and Human Services (DHHS) found that at worksites which offer physical activity programs, employers have been able to reduce healthcare costs by 20–55%, reduce short term sick leave by 6–32%, and increase overall productivity [63]. In October 2006, DHHS launched its project to develop a comprehensive set of guidelines relating to physical activity and nutrition for Americans in a variety of settings [64].

### Insurance Coverage Mandates

A final legal theme in combating America's obesity epidemic focuses on regulations that target insurance practices. Although private insurance companies in the U.S. typically cover the health effects of obesity (e.g., type II diabetes, heart disease), they usually do not cover treatment, through programs or surgeries, of obesity itself. Private health insurers feature different policies and formulas for determining whether they will cover a particular surgery related to morbid obesity [65]. This lack of uniformity prevents some patients from receiving treatment for a highly debilitating condition, primarily because they cannot afford treatment in the U.S. health care system.

Government health care programs, such as the U.S. Medicare program, may provide coverage for select surgeries relating to morbid obesity. Medicare is a federal program that subsidizes health care insurance primarily for Americans age 65 and older and some people with disabilities under the age of 65. A Medicare recipient must meet specific requirements to be eligible for morbid obesity surgery, including having a Body Mass Index (BMI) greater than 35, being diagnosed with at least one concurrent illness related to obesity, and having previously and unsuccessfully attempted to treat obesity through medically-supervised care (and not just personal efforts to, for example, lose weight through diet plans) [66].

Public funding of surgery for morbid obesity underscores the necessity for private insurance companies to offer such coverage as well. Some states have introduced legislation to require insurance providers to cover obesity-specific treatments. For example, the Indiana legislature requires the state's group insurance plan to provide coverage for the treatment of morbid obesity among public employees [67]. The Missouri state legislature amended its laws to mandate that all health benefit plans renewed after August 28, 2007 cover morbid obesity treatment [68]. Furthermore, Idaho's legislature amended its health insurance laws to require coverage for expenses of services associated with morbid obesity [69].

## Conclusion

These legal themes for obesity regulation offer a wide array of interventions to directly or indirectly combat this serious public health threat in the United States. They include efforts that are focused on individual behaviors and norms (e.g., the use of financial disincentives), as well as communal objectives (e.g., zoning laws, school nutrition programs). Actors in public and private sectors are targeted through these laws and policies, consistent with the understanding that protecting the public's health is a shared, societal goal. While protecting the health of populations, particularly children, may be the source of many of these legal provisions, it is necessary to balance other laudable interests and perspectives.

Though capable of illustration, the viability of these legal themes in curbing obesity is uncertain. Measuring the effectiveness of any legal intervention, even ones that are specifically intended to reduce obesity in populations, is challenging. Determining the effects of single or multiple legal interventions is complicated by the need to assess numerous factors that can contribute to, or detract from, a healthier population. Lack of uniformity in the regulations between states and localities limits comparisons among jurisdictions. Implementation of some of these legal themes is at an early stage, thus not allowing for sufficient time to assess their impact, even assuming that adequate studies could be designed to achieve this goal. Increased efforts to study and assess these legal interventions may lead to more refined approaches that are tailored to lowering obesity among the U.S. population.

## Authors' contributions

JGH conceived the conceptual framework and design for the manuscript. JGH, AMG, and SS conducted research concerning the framework for the manuscript. JGH, AMG, and SS participated in the drafting, editing, and approval of the final manuscript.
